# Expert consensus on the diagnosis and management of tooth developmental anomalies

**DOI:** 10.1038/s41368-025-00401-8

**Published:** 2026-01-20

**Authors:** Jingxian Zhu, Mian Wan, Xiaohong Duan, Zhipeng Fan, Yao Sun, Xudong Wang, Shuguo Zheng, Liwei Zheng, Qinglin Zhu, Dong Chen, Jiewen Dai, Dong Han, Miao He, Cui Huang, Yuegui Jiang, Zhonglin Jia, Yihuai Pan, Yongchu Pan, Tiemei Wang, Wenmei Wang, Baoshan Xu, Wei Yin, Tingting Zhang, Yanli Zhang, Zhenjin Zhao, Zhuan Bian, Yaling Song

**Affiliations:** 1 State Key Laboratory of Oral & Maxillofacial Reconstruction and Regeneration, Key Laboratory of Oral Biomedicine Ministry of Education, Hubei Key Laboratory of Stomatology, School & Hospital of Stomatology, Wuhan University, Wuhan, China; 2 Department of Geriatric Dentistry, School & Hospital of Stomatology, Wuhan University, Wuhan, China; 3 State Key Laboratory of Oral Diseases & National Center for Stomatology & National Clinical Research Center for Oral Diseases & Department of Cariology and Endodontics, West China Hospital of Stomatology, Sichuan University, Chengdu, China; 4 State Key Laboratory of Oral & Maxillofacial Reconstruction and Regeneration, National Clinical Research Center for Oral Diseases, Shaanxi Key Laboratory of Stomatology, Department of Oral Biology, Clinic of Oral Rare Diseases and Genetic Diseases, School of Stomatology, The Fourth Military Medical University, Xi’an, China; 5 Laboratory of Molecular Signaling and Stem Cells Therapy, Beijing Key Laboratory for Tooth Regeneration and Function Reconstruction of Oral Tissues, School of Stomatology, Beijing Stomatological Hospital, Capital Medical University, Beijing, China; 6 Department of Implantology, Stomatological Hospital and Dental School of Tongji University, Shanghai Engineering Research Center of Tooth Restoration and Regeneration, Shanghai, China; 7 Department of Oral and Cranio-Maxillofacial Surgery, Shanghai Ninth People’s Hospital, Shanghai Jiao Tong University School of Medicine; College of Stomatology, Shanghai Jiao Tong University; National Center for Stomatology; National Clinical Research Center for Oral Diseases; Shanghai Key Laboratory of Stomatology, Shanghai, China; 8 Department of Preventive Dentistry, Peking University School and Hospital of Stomatology & National Center for Stomatology & National Clinical Research Center for Oral Diseases & National Engineering Research Center of Oral Biomaterials and Digital Medical Devices & Beijing Key Laboratory of Digital Stomatology & NHC Key Laboratory of Digital Stomatology & NMPA Key Laboratory for Dental Materials, Beijing, China; 9 State Key Laboratory of Oral Diseases & National Center for Stomatology & National Clinical Research Center for Oral Diseases & Department of Pediatric Dentistry, West China Hospital of Stomatology, Sichuan University, Chengdu, China; 10 State Key Laboratory of Oral & Maxillofacial Reconstruction and Regeneration, National Clinical Research Center for Oral Diseases, Shaanxi Key Laboratory of Stomatology, Department of Operative Dentistry and Endodontics, School of Stomatology, The Fourth Military Medical University, Xi’an, China; 11 School of Stomatology, Zhengzhou University; Department of Stomatology, the First Affiliated Hospital of Zhengzhou University, Zhengzhou, China; 12 Department of Prosthodontics, Peking University School and Hospital of Stomatology & National Center for Stomatology & National Clinical Research Center for Oral Diseases & National Engineering Research Center of Oral Biomaterials and Digital Medical Devices, Beijing, China; 13 Department of Pediatric Dentistry, School & Hospital of Stomatology, Wuhan University, Wuhan, China; 14 Department of Prosthodontics, School & Hospital of Stomatology, Wuhan University, Wuhan, China; 15 Department of Cariology & Endodontics, College of Stomatology, Xi’an Jiaotong University, Xi’an, China; 16 State Key Laboratory of Oral Diseases & National Center for Stomatology & National Clinical Research Center for Oral Diseases & Department of Cleft Lip and Palate, West China Hospital of Stomatology, Sichuan University, Chengdu, China; 17 Department of Endodontics, School and Hospital of Stomatology, Wenzhou Medical University, Wenzhou, China; 18 State Key Laboratory Cultivation Base of Research, Prevention and Treatment for Oral Diseases & Jiangsu Province Engineering Research Center of Stomatological Translational Medicine & Department of Orthodontics, The Affiliated Stomatological Hospital of Nanjing Medical University, Nanjing, China; 19 Department of Oral Radiology, Nanjing Stomatological Hospital, Affiliated Hospital of Medical School, Institute of Stomatology, Nanjing University, Nanjing, China; 20 Department of Oral Medicine, Nanjing Stomatological Hospital, Affiliated Hospital of Medical School, Institute of Stomatology, Nanjing University, Nanjing, China; 21 Hospital of Stomatology, Guangdong Provincial Key Laboratory of Stomatology, Guanghua School of Stomatology, Institute of Stomatological Research, Sun Yat-sen University, Guangzhou, China; 22 Department of Cariology and Endodontics, School & Hospital of Stomatology, Wuhan University, Wuhan, China; 23 Tianjin Medical University School and Hospital of Stomatology, Tianjin, China; 24 The First Clinic, Orthodontic Department, School and Hospital of Stomatology, China Medical University, Liaoning Province Key Laboratory of Oral Disease, Shenyang, China

**Keywords:** Dental diseases, Dentistry

## Abstract

Tooth developmental anomalies are a group of disorders caused by unfavorable factors affecting the tooth development process, resulting in abnormalities in tooth number, structure, and morphology. These anomalies typically manifest during childhood, impairing dental function, maxillofacial development, and facial aesthetics, while also potentially impacting overall physical and mental health. The complex etiology and diverse clinical phenotypes of these anomalies pose significant challenges for prevention, early diagnosis, and treatment. As they usually emerge early in life, long-term management and multidisciplinary collaboration in dental care are essential. However, there is currently a lack of systematic clinical guidelines for the diagnosis and treatment of these conditions, adding to the difficulties in clinical practice. In response to this need, this expert consensus summarizes the classifications, etiology, typical clinical manifestations, and diagnostic criteria of tooth developmental anomalies based on current clinical evidence. It also provides prevention strategies and stage-specific clinical management recommendations to guide clinicians in diagnosis and treatment, promoting early intervention and standardized care for these anomalies.

## Introduction

Tooth development is a highly orchestrated biological process that begins with the formation of the dental lamina and placode, progressing through distinct stages such as morphogenesis, histodifferentiation, and mineralization.^1^ Each stage relies on precise spatial and temporal signaling interactions between epithelial and mesenchymal tissues.^1^ Disruptions at any point in this process can result in tooth developmental anomalies.^1,2^ These disruptions may arise from genetic factors, such as mutations in genes critical for tooth development, and/or from environmental influences, including exposure to certain chemicals, infections, trauma, and other external factors.^3^ As a result, dental anomalies can present in various forms, including abnormalities in tooth number, size, shape, or structure.^4^

Beyond common infectious diseases, developmental dental anomalies are also significant contributors to the impairment of tooth hard tissues and have a substantial impact on oral health.^5^ These anomalies are rooted in congenital disturbances and cause irreversible damage.^6,7^ Once affected, the compromised hard tissue cannot regenerate, leading to a lifelong impact. They can further negatively affect mastication, articulation, and facial esthetics, thereby reducing quality of life and potentially harming mental health.^5,8^ Due to the low prevalence of tooth developmental anomalies, dentists often have a limited understanding of these anomalies, making it challenging for them to provide standardized diagnoses, clinical management, and appropriate oral health guidance. Therefore, the diagnosis and treatment of tooth developmental anomalies are critical aspects of oral disease prevention and management.

This consensus primarily focuses on tooth developmental anomalies that occur in isolation, including structural anomalies of enamel and dentin, as well as morphological anomalies such as dens invaginatus (DI) and taurodontism. Based on current clinical evidence, this consensus was developed through the collaborative efforts of a multidisciplinary team of dental experts. It provides a comprehensive analysis of the classifications, etiology, clinical manifestations of these anomalies, and diagnostic criteria or proposed diagnostic guidelines, as well as strategies for prevention and clinical management. The goal is to offer guidance for clinicians in diagnosing and managing these conditions effectively. For tooth anomalies associated with systemic syndromes, this expert consensus offers clinical management recommendations specifically for the accompanying structural or morphological abnormalities but does not address other systemic manifestations.

## Common tooth developmental anomalies

Tooth developmental anomalies can arise from disturbances at various stages of odontogenesis, affecting tooth number, morphology, structure, eruption, and exfoliation.^1^ Abnormalities during the initiation stage, such as disruptions in dental placode formation, can result in anomalies of tooth number, including hypodontia, oligodontia, and supernumerary teeth.^9^ Defects during the morphodifferentiation stage lead to morphology-related anomalies such as microdontia, macrodontia, peg-shaped teeth, DI, taurodontism, radix entomolaris, and radix paramolaris.^10^ Alterations during the maturation stage, which governs tooth mineralization, give rise to structural anomalies such as amelogenesis imperfecta (AI), dentinogenesis imperfecta (DGI), and dentin dysplasia (DD).^11^ In addition, abnormalities affecting root formation or bone remodeling may cause abnormal tooth eruption or exfoliation. These anomalies are typically irreversible, with lasting impacts on both the appearance and function of the dentition.^12^ This consensus primarily focuses on the structural and morphological dental anomalies.

### Tooth structural anomalies

Tooth structural anomalies specifically refer to developmental defects in the tooth hard tissues, including enamel, dentin, or cementum. These abnormalities arise from disruptions in the formation or mineralization of these hard tissues and often result in compromised mechanical strength, function, or esthetics.

#### Developmental defects of enamel

Developmental defects of enamel (DDEs), resulting from heritable or environmental factors during enamel formation, can lead to alterations in enamel quantity, composition, and structure. These defects typically present as abnormalities in enamel color, thickness, and mineralization.^4,11,13^

AI has a reported prevalence ranging from 1 in 700 to 1 in 14 000 across different populations.^14,15^ A recent multicenter survey in China involving 1355 patients diagnosed with isolated rare dental diseases identified 628 cases of AI, accounting for 46.35% of the cohort.^16^ According to the classifications proposed by Sundell et al. and Smith et al., AI can be categorized into hypoplastic, hypomaturation, and hypocalcified subtypes.^17,18^ Additionally, AI is frequently associated with syndromes such as tricho-dento-osseous (TDO) syndrome, rickets, ectodermal dysplasia, and some others.^15,19,20^

Environmental developmental defects of enamel (eDDEs) result from systemic or local factors, including malnutrition, chemical exposure, medications, infections, or trauma, occurring during the prenatal, perinatal, or postnatal periods.^4,11,21^ Typical anomalies within this category include dental fluorosis, tetracycline-stained teeth, Turner’s hypoplasia, and molar-incisor hypomineralization (MIH), and other related conditions.^4^

#### Developmental defects of dentin

Developmental defects of dentin (DDDs) refer to abnormalities in dentin structure and function, manifesting as changes in dentin morphology, density, hardness, color, and luster.^22^ These defects are primarily attributed to heritable factors and can affect the tooth’s appearance, hardness, sensitivity to stimuli, and overall health.^23^

According to Shields’ classification, hereditary dentin defects include DGI types I to III and DD types I and II.^24^ DGI commonly affects both the crowns and roots (including pulp chambers and root canals) of teeth, while DD primarily impacts the morphology of the roots, pulp chamber, and root canals. The reported prevalence of DGI-II is estimated to range from 1 in 8 000 to 1 in 6 000 (ref. ^14^), whereas DD-I is considerably rarer, with a prevalence of approximately 1 in 100 000 (ref. ^25^). Hereditary dentin defects can occur either in isolation or in association with systemic diseases. The associated systemic diseases are primarily bone disorders, due to the shared regulatory roles of the causative genes in both dentin and bone development.^26^ Representative examples include osteogenesis imperfecta (whose oral manifestation is referred to as DGI-I) and rickets.^26^

DDDs caused by environmental factors are rarely observed. Molar-incisor malformation (MIM) is potentially linked to systemic conditions. It is characterized by severe root malformations predominantly affecting molars and incisors.^27,28^ The exact prevalence of MIM remains uncertain.

#### Developmental defects of cementum

Developmental defects of cementum (DDC) are rare conditions that can affect the structure, thickness, mineralization, and attachment of cementum to surrounding tissues. These defects are generally categorized into cementum hypoplasia and cementum hyperplasia.^4^ Certain systemic diseases, such as hypophosphatasia and rickets, are occasionally associated with cementum defects.^29,30^ Due to the limited documentation and case reports available, this consensus does not include detailed discussions on DDCs.

### Tooth morphological anomalies

Developmental morphological tooth anomalies include conditions such as DI, taurodontism, central accessory cusps, fused teeth, geminated teeth, concrescence, microdontia, macrodontia, radix entomolaris, radix paramolaris, and others. This consensus primarily focuses on morphological anomalies that present significant challenges in diagnosis and treatment, with a particular focus on DI and taurodontism.

#### Dens invaginatus

DI results from the abnormal invagination of the dental epithelium into the mesenchyme, typically leading to the folding of enamel into dentin.^31^ The global prevalence of DI ranges from 0.04% to 10%,^32^ with an estimated prevalence of approximately 3.0% in the Chinese population.^33^ Based on the site of invagination, DI is classified into coronal DI (CDI) and radicular DI (RDI).^31^ The widely adopted Oehlers classification further categorizes CDI into types I, II, and III according to the depth of the invagination from the crown toward the apex.^34^ In contrast, RDI, which is less common than CDI, results from the invagination of Hertwig’s epithelial root sheath (HERS) into the developing tooth root.^35^

#### Taurodontism

The failure of horizontal elongation of HERS at the appropriate coronoapical level of the tooth can result in taurodontism.^36^ This morphological anomaly is characterized by an enlarged pulp chamber extending apically, an absent or apically displaced bottom of the pulp chamber, and an elongated root trunk. Additional features commonly include apically positioned root furcation, shortened tooth roots, and a lack of significant constriction at the cementoenamel junction (CEJ).^36,37^ Taurodontism can occur as an isolated condition or as a concomitant feature of various syndromes.^38^ The reported prevalence of taurodontism varies widely, ranging from 0.1% to 48%, due to differences in diagnostic criteria and studied populations.^36^

## The etiology of tooth developmental anomalies

### Genetic factors

Tooth development is regulated by thousands of genes, and mutations in any of these genes would lead to functional disruptions, resulting in developmental defects.^1^ These genetic mutations may occur de novo, arising spontaneously without parental inheritance, or may be inherited from one or both parents.^14,25^

Tooth developmental anomalies, such as AI, DGI, and DD, have been linked to specific causative genes, most of which follow Mendelian inheritance patterns. The identified genes and their corresponding inheritance modes are summarized in Table 1. AI can manifest either as an isolated condition, termed non-syndromic AI, or as a concomitant feature of a syndromic disorder.^39^ Table 1 specifically addresses non-syndromic AI. To date, 19 syndromic disorders associated with AI and 27 causative genes have been documented in the *Online Mendelian Inheritance in Man* (OMIM) database (https://omim.org/). Among these syndromes, nephrocalcinosis syndrome (also known as enamel-renal syndrome) caused by *FAM20A* mutations, is particularly notable. This condition is characterized by a distinct oral phenotype, including enamel hypoplasia.^40,41^ *FAM20A* mutation is one of the most frequently reported mutations in syndromic AI.^42^

**Table 1:** Common causative genes and their inheritance patterns in non-syndromic AI, DGI, and DD

Anomaly	Type	Causative gene and inheritance pattern
AI (non-syndromic)	Hypoplastic	*ACP4* (AR),^151^ *AMBN* (AR),^152^ *AMELX* (XLD),^153^ *ENAM* (AD/AR),^154,155^ *ITGB6* (AR),^156^ *LAMA3* (AD),^157^ *LAMB3* (AD),^158^ *SP6* (AD)^159^
	Hypomaturation	*GPR68* (AR),^160^ *KLK4* (AR),^161^ *MMP20* (AR),^162^ *SLC24A4* (AR),^163^ *WDR72* (AR)^164^
	Hypocalcified	*AMTN* (AD),^165^ *FAM83H* (AD),^166^ *RELT* (AR)^167^
DGI	Type I	*COL1A1* (AD),^168^ *COL1A2* (AD)^169^
	Type II	*DSPP* (AD)^6^
	Type III	*DSPP* (AD)^170^
DD	Type I	*SMOC2* (AR),^171^ *SSUH2* (AD),^172^ *VSP4B* (AD)^173^
	Type II	*DSPP* (AD)^6^

*AI* amelogenesis imperfecta, *DGI* dentinogenesis imperfecta, *DD* dentin dysplasia. *AD* Autosomal dominant inheritance, *AR* Autosomal recessive inheritance, *XLD* X-linked dominant inheritance. The table includes only the common causative genes, with their corresponding inheritance patterns indicated in parentheses. The causative genes were listed alphabetically. Representative literature references are provided for each entry

Certain tooth developmental anomalies have been reported to be associated with genetic factors; however, the specific causative genes have not yet been fully identified. For instance, DI may be linked to mutations in *KIF4A* and *ADAR1.*^43,44^ Taurodontism is commonly associated with hereditary systemic diseases,^37^ such as odonto-onycho-dermal dysplasia caused by *WNT10A* mutation.^45^ Recent studies have elucidated the causal relationship between *Wnt10a* mutation and taurodontism.^46,47^ However, taurodontism can also occur as an isolated condition without systemic manifestations.^48^ The pathogenic mechanisms underlying non-syndromic taurodontism, however, remain largely unknown. With the rapid advancement of genetic testing technologies and experimental research, the identification of additional causative genes is approaching, thereby improving our understanding of these anomalies and providing valuable insights for their prevention and treatment.

### Environmental factors

External influences, distinct from intrinsic genetic factors, are collectively referred to as environmental factors. During tooth development, these factors could affect various stages, leading to structural and morphological anomalies.^11^ Based on their scope of impact, environmental factors are further categorized into systemic factors and local insults.

#### Systemic factors

Systemic factors can influence tooth development across the prenatal, perinatal, and postnatal periods. For example, prenatal exposure to tobacco or vitamin D deficiency has been associated with DDE in newborns.^49,50^ Hutchinson’s teeth, a well-documented example of prenatal dental anomalies, result from maternal infection with *Treponema pallidum.*^51^ Perinatal factors, such as premature birth and low birth weight, are significant risk factors for DDE.^52^ The prevalence of DDE in primary dentition is reported to range from 40% to 70% among children born prematurely or with low birth weight.^53^

Postnatal conditions that disrupt calcium-phosphorus metabolism, such as hypocalcemia, hyperbilirubinemia, gastrointestinal disorders, and hypoparathyroidism, can contribute to tooth developmental anomalies.^54–56^ Additionally, inappropriate use of certain medications or exposure to chemicals may interfere with tooth development, as exemplified by tetracycline-stained teeth and dental fluorosis.^57,58^ Systemic infections, including otitis media and viral infections such as varicella-zoster and mumps, have also been implicated in DDE affecting both primary and permanent dentitions.^56,59^

The etiology of MIH and MIM remains unclear. However, evidence suggests potential associations with factors such as antibiotic use, hormonal fluctuations, premature birth, and low birth weight, which may influence specific stages of tooth development.^60,61^ Additionally, maternal mental illnesses like depression or anxiety are associated with MIH.^62^

Currently, only a portion of the mechanisms underlying the effects of systemic environmental factors on tooth development have been elucidated. The associations between most of these factors and tooth developmental anomalies have been identified primarily through clinical case reports and epidemiological studies.^55,63,64^ Recent studies suggest that environmental factors may influence tooth development through epigenetic regulation.^65^ Researches are required to further establish definitive causal relationships and enhance our understanding of these underlying mechanisms.

#### Local factors

Local factors influencing tooth development primarily include trauma and localized infections. When these factors affect developing permanent teeth, they could lead to defects known as Turner’s hypoplasia.^66^ For instance, damage to the alveolar crest caused by laryngoscopy in neonates may result in localized developmental defects in the incisors.^67^ Similarly, traumatic intrusion of primary teeth could disrupt the development of their permanent successors.^68^ In the canine and premolar regions, severe infections of primary teeth are the most common cause of Turner’s hypoplasia.^69^

## Clinical manifestations and diagnoses of tooth developmental anomalies

This section provides the clinical manifestations and diagnoses of tooth developmental anomalies that are relatively prevalent and often associated with significant diagnostic and therapeutic challenges. Specifically, it covers structural anomalies involving enamel and dentin, as well as morphological conditions including dens invaginatus and taurodontism.

### Developmental defects of enamel

#### Amelogenesis imperfecta

##### Clinical manifestations

**Fig. 1:**
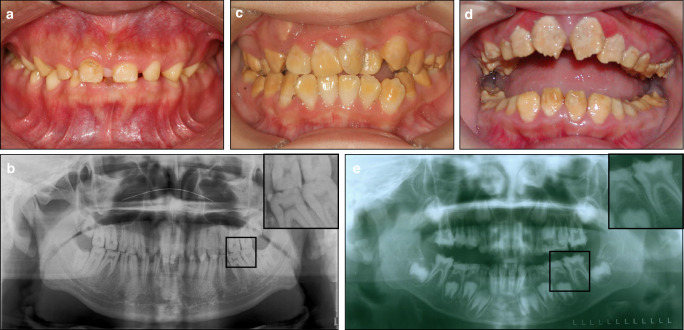
Amelogenesis imperfecta (AI). **a**, **b** Hypoplastic AI in a 26-year-old male. **a** The patient exhibits a generalized reduction in enamel thickness, leading to widely spaced teeth and a lack of proximal contact. **b** The radiograph reveals insufficient enamel thickness. **c** Hypomaturation AI in a 13-year-old female. The patient shows uneven yellow discoloration of the enamel. **d**, **e** Hypocalcified AI in a 9-year-old male. d The patient presents with a rough enamel surface covered with scales. **e** The radiograph shows reduced contrast between the enamel and dentin

Hypoplastic: The enamel in this subtype exhibits normal hardness but reduced thickness.^70,71^ A generalized reduction in enamel thickness may lead to widely spaced teeth with a lack of proximal contact (Fig. 1a), whereas localized reductions often present as pits or grooves on the enamel surfaces.^72,73^ Although the enamel is thinner, its color and mineralization remain normal. Radiographically, the contrast between enamel and dentin appears normal, although the enamel layer is notably thin (Fig. 1b).

Hypomaturation: This subtype is characterized by enamel with normal thickness but reduced mineralization, making it susceptible to attrition.^11^ The enamel often exhibits uneven coloration, with yellow or brown discoloration that compromises esthetics (Fig. 1c). On radiographs, the reduced mineralization diminishes the contrast between enamel and dentin, making being difficult to distinguish enamel from dentin.^70^

Hypocalcified: In this subtype, the enamel maintains normal thickness but exhibits reduced hardness, making it more susceptible to attrition and hypersensitivity to thermal or cold stimuli.^11,70^ The enamel surface is typically rough, often pigmented, and covered with scales (Fig. 1d). Additionally, the reduced mineralization further complicates the distinction between enamel and dentin on radiographs (Fig. 1e).

Hypomaturation-hypoplastic with taurodontism: This subtype is characterized by a concurrent presentation of hypomaturation and hypoplastic defects, which may involve the entire dentition or be localized to specific regions. Taurodontism is commonly observed in the molars.^70^

Diagnosis: (1) Intraoral examination reveals alterations in crown color and translucency, frequently accompanied by enamel defects such as pits, grooves, and loss of proximal contact;^71^ (2) Radiographic findings may show either enamel of normal thickness but reduced contrast against dentin or enamel of reduced thickness but normal contrast. The pulp chambers and root canals typically appear normal, with no evidence of narrowing or obliteration;^70,71^ (3) Family history is often present, consistent with autosomal dominant (AD), autosomal recessive (AR), or X-linked inheritance patterns;^42^ (4) Auxiliary genetic testing frequently identifies causative mutations in genes such as *ENAM*, *AMELX*, *MMP20*, *WDR72*, *FAM83H*, *KLK4*, *AMBN*, and others;^39,42^ (5) Environmental factors contributing to DDE should be ruled out to confirm a genetic etiology.

#### Dental fluorosis

**Fig. 2:**
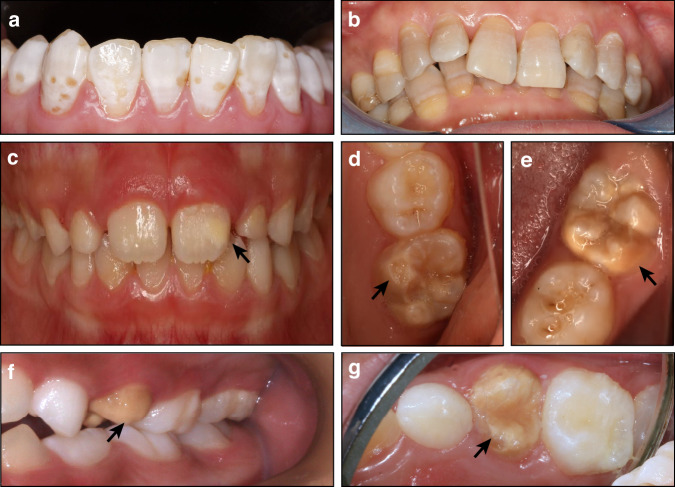
Environmental developmental defects of enamel. **a** Dental fluorosis in a 31-year-old female, presenting as chalky white patches on the enamel surface, accompanied by spotty enamel defects. **b** Tetracycline-stained teeth in a 49-year-old male, characterized by stripped yellow to grey enamel pigmentation. **c**–**e** Molar-incisor hypomineralization in a 7-year-old male. **c** The 21 shows creamy discoloration of the enamel. **d** The 26 displays enamel breakdown with well-defined margins (black arrow). **e** The 36 shows yellow discoloration with defined margins (black arrow). **f**, **g** Turner’s hypoplasia in a 10-year-old male at the site of 24 (black arrow), demonstrating yellow discoloration and irregular crown morphology

Clinical manifestations: The enamel presents with localized discoloration, ranging from chalky white to brown (Fig. 2a). In severe cases, enamel defects may also be present.^74,75^ This condition typically affects groups of teeth that erupt during the same developmental period, depending on the timing and duration of exposure to elevated fluoride levels. Permanent teeth are most commonly affected, exhibiting increased resistance to acid yet heightened susceptibility to wear.^74^

Diagnosis (adapted criteria from the standard of National Health Commission of the People’s Republic of China:^76^) (1) Patients have a clear history of high fluoride exposure during tooth development, such as residing in regions with elevated fluoride concentrations; (2) Examination of the enamel surface after drying exhibits discoloration ranging from chalky white to brown patches that cannot be removed. In severe cases, the enamel structural defects may be present, varying from spotty or honeycomb-like patterns to map-like appearances.

#### Tetracycline-stained teeth

Clinical manifestations: The enamel exhibits discoloration, typically presenting as yellow, gray, or brown pigmentation (Fig. 2b). In mild cases, the discoloration appears uniform, whereas severe cases exhibit with striped patterns of pigmentation.^77^

Diagnosis: (1) History of maternal or childhood tetracycline use; (2) Enamel discoloration ranging from yellow to gray or brown, which may present as uniform staining or in striped patterns.^77^

#### Molar-incisor hypomineralization

Clinical manifestations: MIH is characterized by localized opacities on the surface of molars and incisors, typically sparing the cervical region (Fig. 2c–e). The discoloration varies in severity, ranging from white or creamy to yellow and brown.^78^ During the early stages of tooth eruption, enamel thickness remains normal, with alterations limited to changes in coloration. However, post-eruptive mastication can induce enamel breakdown (Fig. 2d), exposing the underlying dentin and significantly increasing the susceptibility to dental caries.^79^

Diagnosis (adapted criteria from the European Association of Pediatric Dentistry:^79^) (1) Enamel hypomineralization affecting at least one of the first permanent molars, with possible involvement of the incisors; (2) The presence of opacities with well-defined margins, typically exceeding 1 mm in diameter; (3) Post-eruptive enamel breakdown, which may rapidly progress to caries; (4) Hypersensitivity in affected teeth; (5) It may present as atypical restorations with the size and shape deviating from common carious lesions. These restorations may extend to surfaces less susceptible to caries, or opacities with well-defined margins may be observed at the restoration margins; (6) Tooth loss attributed to MIH.

#### Turner’s hypoplasia

Clinical manifestations: Turner’s hypoplasia typically affect a single permanent tooth, presenting with white or brown discolorations on the enamel surface, enamel defects, and even irregular crown formation (Fig. 2f, g). The predilection sites for Turner’s hypoplasia are the premolars and central incisors.

Diagnosis: (1) A history of trauma or infection affecting the primary predecessor tooth; (2) Discoloration or enamel defects localized to a single permanent tooth.^66,80^

#### Differential diagnosis of developmental defects of enamel

To establish a differential diagnosis of DDEs, a comprehensive assessment is required, including the patient’s family history, dental and systemic health history, and residency history, in addition to characteristic clinical manifestations. Genetic testing may be recommended when necessary. The key considerations for the differential diagnosis of DDEs are summarized in Table 2.

**Table 2:** Summary of clinical features of developmental defects of enamel

Anomaly	Characteristic manifestations	Affected teeth	Radiographic feature	Hereditary	Patient history
Amelogenesis imperfecta	Normal hardness but reduced thickness, or normal thickness but reduced hardness. Normal color or yellow/brown discoloration. Smooth surface or with defects.	Primary and permanent dentitions. All teeth.	Reduced enamel thickness or low contrast with dentin	Yes	None
Dental fluorosis	Chalky white to brown patches, possibly with enamel defects.	Mainly permanent dentition. All teeth or simultaneously erupted teeth.	-	No	Exposure to high levels of fluoride
Tetracycline-stained teeth	Striped discoloration in varying degrees of yellow, grey, or brown.	Mainly permanent dentition. All teeth.	-	No	History of tetracycline use
Molar-incisor hypomineralization	Normal enamel thickness with opaque patches of distinct margins. Enamel loss after eruption.	Mainly permanent dentition. Molars and incisors.	Atypical caries	No	History of childhood diseases
Turner’s hypoplasia	White or brown patches with enamel defects.	Mainly permanent teeth. Individual teeth (commonly premolars)	-	No	Infection or trauma of precursor primary teeth

“-” indicates no characteristic radiographic features

### Developmental defects of dentin

#### Dentinogenesis imperfecta type I

Clinical manifestations: DGI-I is a dental phenotype associated with osteogenesis imperfecta, a systemic disorder characterized by bone abnormalities. Both primary and permanent dentitions can be affected, although the severity of involvement may vary among teeth. Affected teeth typically appear translucent with an amber-like discoloration. The enamel is fragile, prone to fractures, and often lost by the time of clinical evaluation, exposing the underlying dentin, which is usually severely worn. Systemic manifestations include skeletal fragility, with osteoporosis predisposing individuals to fractures, which may be accompanied by deformities of the appendicular skeleton and spine. The sclera may appear blue or maintain a normal coloration. Radiographic findings commonly include obliteration of the dental pulp and short, narrow roots.^23,81^

Diagnosis: (1) The patient has a confirmed diagnosis of osteogenesis imperfecta; (2) Both primary and permanent teeth exhibit translucency with an amber-like discoloration and significant wear; (3) Radiographic imaging typically shows obliterated pulp chambers and root canals. Short or narrowed roots may also be present. Notably, within a single individual, both normal and obliterated pulp chambers and canals may be observed; (4) The patient typically presents with a family history consistent with an autosomal dominant inheritance pattern; (5) Auxiliary genetic testing usually identifies mutations in genes such as *COL1A1*, *COL1A2*, and others.^23–25^

#### Dentinogenesis imperfecta type II

**Fig. 3:**
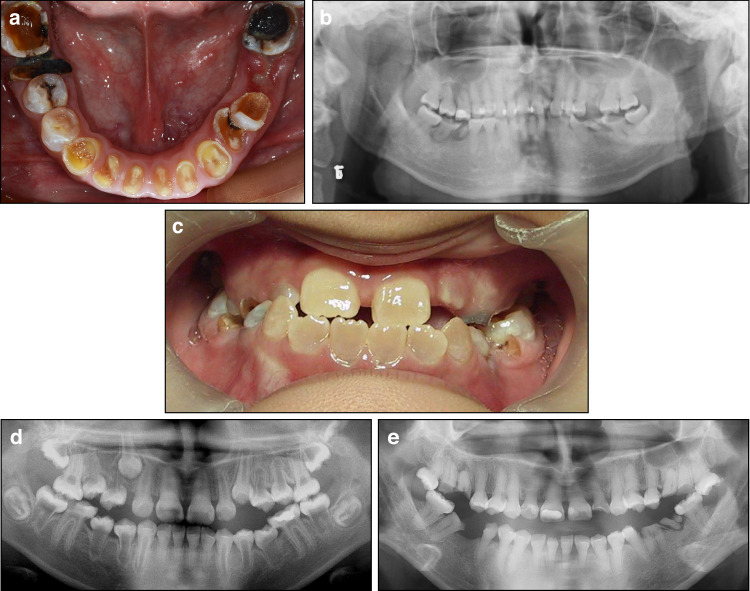
Dentinogenesis imperfecta (DGI). **a**, **b** DGI-II in a 32-year-old male. **a** The crowns are severely worn, exposing the amber-colored dentin beneath. **b** The radiograph reveals obliterated pulp chambers and root canals and constricted cervical regions. **c**–**e** DGI-III. **c** A 12-year-old male proband at the first visit, showing amber-colored teeth and attrition of the posterior teeth. **d** The radiograph demonstrates the DGI-III shell-teeth phenotype in the canines and posterior teeth, while the earlier-erupted teeth exhibit obliterated pulp chambers and root canals characteristic of DGI-II. **e** A follow-up radiograph taken at 29 years of age for the same patient shows complete obliteration of the pulp chambers and root canals across the entire dentition, indicating progression from the DGI-III phenotype to the DGI-II phenotype

Clinical manifestations: DGI-II, also known as hereditary opalescent dentin, affects both primary and permanent dentition. The condition is characterized by a bluish-gray opalescence of the teeth. The enamel is highly susceptible to wear and loss, exposing the underlying amber-colored dentin (Fig. 3a). A distinct cervical constriction often leads to a ball-like appearance of the tooth crown.^81,82^ While there are no associated signs of osteogenesis imperfecta, some individuals may present with sensorineural hearing loss.^82,83^ Radiographs typically show either narrowed or completely obliterated pulp chambers and root canals (Fig. 3b).

Diagnosis: (1) Both primary and permanent dentitions can be affected, with the teeth exhibiting bluish-gray or opalescence appearance. Varying degrees of wear leads to enamel loss and the exposed underlying amber-colored dentin; (2) Radiographic imaging reveals obliterated pulp chambers, root canals and cervical constriction; (3) The patients typically do not show signs of osteogenesis imperfecta, although a few may exhibit sensorineural hearing loss; (4) Patients often present with a family history of an autosomal dominant inheritance pattern; (5) Auxiliary genetic testing often identifies mutations in dentin sialophosphoprotein (*DSPP)* gene.^6,84^

#### Dentinogenesis imperfecta type III

Clinical manifestations: Both primary and permanent dentitions can be affected by DGI-III, which exhibits oral manifestations similar to those of DGI-II,^23^ with enamel prone to wear and exposed dentin amber discoloration (Fig. 3c). However, unlike DGI-II, the dentin layer in DGI-III is very thin. Both primary and permanent teeth undergo rapid coronal wear after eruption, often leading to pulp exposure. Radiographic findings typically include enlarged pulp chambers and root canals. A thin layer of dentin underlying the enamel and cementum gives the teeth a characteristic “shell-teeth” appearance.^23,85^

Our research team found that within the same family, the younger patient exhibited the shell-teeth phenotype characteristic of DGI-III, whereas the adult patient presented with obliterated pulp chambers and root canals, the typical phenotype of DGI-II.^86^ In addition, longitudinal follow-up of the younger patient with the shell-teeth phenotype revealed a gradual obliteration of the pulp chambers and root canals in the anterior teeth that erupted earlier (Fig. 3d), with complete obliteration of pulp chambers and root canals in all teeth observed in adulthood (Fig. 3e). These findings suggest that the shell-teeth phenotype of DGI-III may represent only an early-stage manifestation during the initial eruption of permanent teeth. As development progresses, the condition ultimately presents as the obliteration phenotype characteristic of DGI-II. Therefore, we suggest that DGI-III should be considered an early-stage phenotype of DGI-II rather than a distinct classification. Among DGI-II patients, most patients present with obliterated pulp chambers and root canals at the time of tooth eruption, while a few exhibit a transitional progression from DGI-III to DGI-II.

Diagnosis: (1) The tooth crowns exhibit amber or grayish-yellow discoloration. The enamel is lost, and the underlying dentin is rapidly worn, often resulting in the exposure of multiple pulp chambers or root canals; (2) Radiographic imaging reveals enlarged pulp chambers and root canals, characteristic of shell-teeth phenotype. As the patient ages, these enlarged pulp chambers and root canals progressively narrow and eventually become obliterated; (3) Patients often have a family history, with an autosomal dominant inheritance pattern; (4) Auxiliary genetic testing usually identifies mutations in *DSPP* gene.^84,86^

#### Dentin dysplasia type I

**Fig. 4:**
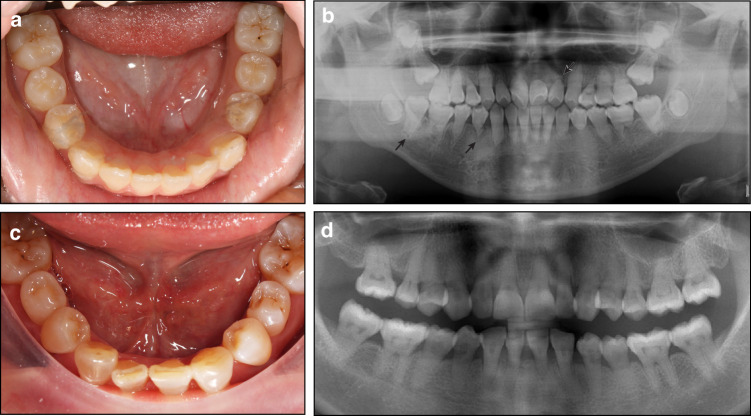
Dentin dysplasia (DD). **a**, **b** DD-I in a 13-year-old female. **a** No obvious abnormalities were observed in the appearance of the teeth. **b** The radiograph shows short, tapered tooth roots, with the pulp chambers obliterated in all teeth except for the crescent-shaped pulp chamber of tooth 24. No root canal images were observed. Black arrows showed the representative periapical radiolucencies of the non-carious teeth. **c**, **d** DD-II in a 33-year-old female. **c** The teeth exhibit slight yellow discoloration. **d** The radiograph reveals obliterated root canals and thistle tube-shaped pulp chambers with pulp stones

Clinical manifestations: Both primary and permanent dentitions can be affected.^82,87^ Clinically, the oral appearance is often unremarkable due to the normal color, shape, and morphology of the tooth crowns (Fig. 4a). However, the roots are markedly shortened and abruptly narrowed, a condition commonly referred to as “rootless teeth”.^88^ Affected teeth exhibit increased mobility and premature exfoliation. Radiographic imaging typically reveals crescent-shaped or absent pulp chambers, along with short, tapered, or completely absent tooth roots (Fig. 4b). Root canals are typically unidentifiable on radiographs, while periapical radiolucencies of non-carious teeth are frequently observed.^23^ Additionally, DD may be accompanied by bone sclerosis in other skeletal regions.^89^

Diagnosis: (1) The teeth maintain a normal shape and color, but malocclusion may be present; (2) The affected teeth usually exhibit varying degrees of mobility and premature loss; (3) Radiographic findings include short, tapered, or absent tooth roots, crescent-shaped or completely obliterated pulp chambers, and periapical radiolucencies of non-carious teeth; (4) Affected individuals frequently have a family history consistent with an autosomal dominant inheritance pattern.^23,81^

#### Dentin dysplasia type II

Clinical manifestations: The primary teeth exhibit features similar to DGI-II, including significant attrition, exposed amber-colored dentin, and obliterated pulp chambers and root canals.^23,90^ In contrast, the permanent teeth typically appear nearly normal or may exhibit only slight discoloration (Fig. 4c). Radiographic imaging reveals obliterated root canals and thistle tube-shaped coronal pulp chambers that may contain pulp stones (Fig. 4d) or shows pulp obliteration occasionally.^91^ Our group uncovered a distinctive X-shaped root canal phenotype in young permanent teeth, which narrows at the middle of the canal.^92^ This pattern is likely indicative of the initiation of abnormal mineral deposition at the middle of the canal.^93^

Diagnosis: (1) The primary dentition is characterized by significant attrition and exposed amber-colored dentin, with radiographic imaging typically revealing obliterated pulp chambers and root canals; (2) The permanent dentition generally appears nearly normal, with no significant attrition or discoloration, which may increase the risk of misdiagnosis. Radiographic findings include thistle tube-shaped coronal pulp chambers, pulp stones, obliterated root canals, or occasional obliterated pulp chambers; (3) Patients often have a family history consistent with an autosomal dominant inheritance pattern; (4) Genetic analysis commonly identifies mutations in *DSPP* gene as an auxiliary diagnostic marker.^92^

#### Molar-incisor malformation

Clinical manifestations: The defining feature of MIM is the severe underdevelopment of the roots of the permanent first molars. Approximately half of the cases also exhibit root anomalies in the primary second molars and crown abnormalities in the primary incisors, typically presenting as a wedge-shaped defect in the cervical third of the crown.^27,94^ The remaining teeth are rarely affected, and the molar crowns generally appear normal. Radiographic examination typically reveals narrowed, crescent-shaped pulp chambers, along with significantly shortened, tapered, or even absent tooth roots.^27,95^

Diagnosis: (1) The molar crowns appear normal; however, wedge-shaped defects may be observed in the cervical third of the tooth crown; (2) Radiographic features include a narrowed cervical region in the molars, crescent-shaped pulp chambers, and short, tapered tooth roots.^27,94^

#### Differential diagnosis of developmental defects of dentin

The differential diagnosis of DDDs primarily relies on characteristic oral manifestations and radiographic findings, with genetic testing serving as an auxiliary diagnostic tool when necessary. Key distinguishing features of DDDs are summarized in Table 3.

**Table 3:** Summary of clinical features of developmental defects of dentin

Characteristic features	DGI-I	DGI-II	DGI-III	DD-I	DD-II	MIM
Systemic manifestation						
Bone abnormalities, blue or normal sclera	√	-	-	-	-	-
Oral manifestation						
Attrition and loss of enamel	√	√	√	-	√1	-
Amber-colored dentin	√	√	√	-	√1	-
Radiographic features						
Constriction of the cervical region and ball-like crown	√	√	√	-	√1	-
Obliterated pulp chambers and root canals	√	√	-	√	√1	-
Obliterated root canals and thistle tube-shaped pulp chambers with pulp stones	**-**	**-**	**-**	**-**	√2	-
Shell-teeth	**-**	**-**	√	**-**	**-**	**-**
Crescent-like pulp chamber	**-**	**-**	**-**	√	**-**	√
Short, constricted, tapered, absent, or other root anomalies	+/-	**-**	**-**	√	**-**	√
Periapical radiolucency	√	√	√	√	**-**	+/-
Tooth-type specificity (permanent first molar, and/or second primary molar, and/or permanent incisor)	-	-	-	-	**-**	√

*DGI* dentinogenesis imperfecta, *DD* dentin dysplasia, *MIM* molar-incisor malformation. “-” indicates that the anomaly typically does not exhibit this feature. “√” indicates the presence of the feature in the anomaly. “+/-” indicates that the manifestation occurs inconsistently and is observed only in a subset of cases. “1” refers to features observed in the primary dentition. “2” refers to features observed in the permanent dentition

### Tooth morphological anomalies

#### Dens invaginatus

**Fig. 5:**
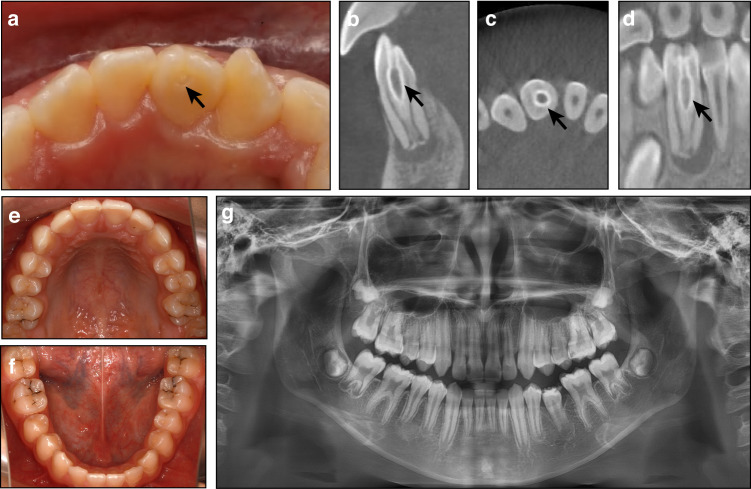
Tooth morphological anomalies. **a**–**d** Dens invaginatus. (a) The crown of tooth 41 displays a palatal pit (black arrow). **b**–**d** Cone-beam computed tomography reveals a sac-like radiolucency surrounded by a high-density radiopaque border (black arrows). **e**–**g** Taurodontism in a 13-year-old male. **e**, **f** The external morphology of the teeth appears normal. **g** The radiograph shows apically positioned pulp chamber floors in the molars

Clinical manifestations: The crown morphology of affected teeth is variable, often presenting with a palatal pit or groove (Fig. 5a). In some cases, the teeth may exhibit a barrel-shaped or conical appearance.^31^ A minority of affected teeth may exhibit with a labial groove. Additionally, crown size may be either larger or smaller compared to their homologous teeth.^31^ DI most frequently occurs in the maxillary lateral incisors, followed by the maxillary central incisors.^31^

According to Oehlers’ classification, CDI includes three distinct types based on the extent and location of the invagination.^31,96^ Type I invagination is confined to the crown and does not extend beyond the CEJ. In Type II, the invagination extends beyond the CEJ into the root but does not reach the apex. It forms a blind sac, which may communicate with the pulp while remaining unconnected to the periodontal ligament. In Type III, the invagination extends through the entire root and establishes a direct communication with the periodontal ligament.

Unlike CDI, RDIs originate from the tooth root and do not involve the crown, with diagnosis primarily dependent on radiographic evaluation.^97^ Type I RDI is characterized by axial infoldings of the root surface, also referred to as a developmental radicular groove. Type II RDI is defined by a cyst-like invagination originating from the root surface.^97^

Diagnosis: The diagnostic process for CDI begins with an assessment of abnormal crown morphology, which may provide initial clinical clues. However, a definitive diagnosis necessitates radiographic imaging, particularly cone-beam computed tomography (CBCT), to obtain for a detailed assessment. Radiographic findings typically reveal sac-like or groove-like radiolucency surrounded by a high-density radiopaque border (Fig. 5b–d). In Type III CDI, the invagination may mimic a root canal on radiographs. When centrally positioned within the main root canal, the invagination often produces the characteristic “tooth within a tooth” appearance.^31,34,97^

#### Taurodontism

Clinical diagnosis: Clinically, teeth affected by taurodontism often exhibit external morphology similar to that of normal teeth (Fig. 5e, f). The condition can affect both primary and permanent dentitions, though it is less frequently observed in the primary dentition. Mandibular molars are more commonly involved than maxillary molars.^36^ Radiographic examination typically reveals an apically displaced pulp chamber floor and root furcation, along with a coronoapically elongated pulp chamber (Fig. 5g). Taurodontism may present as an isolated dental anomaly; however, it is also commonly associated with genetic disorders such as AI, osteogenesis imperfecta, TDO syndrome, and Ellis-van Creveld syndrome.^36,37^

Diagnosis: Taurodontism is primarily diagnosed based on its characteristic radiographic features. It is essential to recognize that this condition is frequently associated with various genetic conditions,^36,70^ necessitating a thorough evaluation during the diagnostic process.

## Preventions and treatment of tooth developmental anomalies

Raising awareness and improving understanding of these anomalies among both dentists and patients is essential for optimal prevention and management. Early screening and accurate diagnosis are critical, followed by specialized treatment and routine monitoring. Comprehensive care should commence in childhood and be maintained throughout the patient’s lifetime to ensure long-term oral health and function.

### Prevention of tooth developmental anomalies

#### Genetic counseling and genetic diagnosis

Genetic counseling plays a pivotal role in the management of heritable tooth developmental anomalies, particularly those associated with well-established inheritance patterns and pathogenic genes, such as AI and DGI.^98,99^ Grounded in expert genetic knowledge and clinical evidence, genetic counseling provides essential guidance on disease risk assessment, prevention, and management.^100^ By facilitating a comprehensive understanding of the disorder, its hereditary mechanisms, and potential health implications, genetic counseling empowers individuals to make informed decisions regarding their healthcare and reproductive planning.^101^

A precise genetic diagnosis significantly enhances the efficacy of genetic counseling by enabling the development of personalized intervention strategies for individuals with confirmed causative gene mutations.^102^ These strategies facilitate the early detection and management of hereditary tooth anomalies, minimizing their effects on oral and overall health.^103^ For individuals seeking to prevent the transmission of these conditions, in-vitro fertilization (IVF) with pre-implantation genetic diagnosis (PGD) may be considered, provided it aligns with ethical regulations.^99,104^ By proactively addressing these conditions, genetic counseling plays a crucial role in improving health outcomes and enhancing quality of life.^101^

#### Prevention against negative environmental factors

Preventing tooth developmental anomalies caused by environmental factors requires a comprehensive, multi-tiered approach, categorized into prenatal, perinatal, and postnatal stages based on the child’s developmental timelines.

During pregnancy, regular prenatal check-ups are essential for monitoring maternal and fetal health. Comprehensive health assessments should be conducted to ensure adequate nutritional intake, particularly of key nutrients such as vitamin D and calcium, which are vital for tooth development.^49,50^ Expectant mothers should also avoid smoking, alcohol consumption, and medications that may negatively impact tooth formation.^49,50^ Managing appropriate weight gain and preventing infections helps lower the risk of preterm birth and low birth weight, both of which are associated with impaired tooth development.^105^ It is also essential to maintain optimal maternal mental health during pregnancy to reduce the risk of tooth developmental anomalies in offspring.^62^

Early screening and timely intervention in neonates are crucial for ensuring proper tooth development. A comprehensive health assessment should be performed at birth to identify systemic conditions that may adversely affect dental development, such as hypocalcemia and hyperbilirubinemia.^54–56^ Monitoring key elements, including calcium and phosphorus, is essential, as these elements play a pivotal role in tooth mineralization.^54–56^ Furthermore, neonates and young children should be protected from viral infections and episodes of hyperpyrexia, as these factors can further compromise normal odontogenesis.^56,59^

To prevent dental fluorosis, the fluoride intake must be carefully regulated. Maintaining an optimal fluoride concentration in drinking water is the most effective preventive measure, ensuring the caries-preventive benefits of fluoride while minimizing the risk of overexposure.^106,107^

Postnatal care plays a crucial role in ensuring normal tooth development and requires attention to several key factors. Alveolar bone trauma should be carefully avoided, as it can significantly impact the development of permanent teeth. In cases of trauma to primary teeth, prompt dental evaluation and treatment are essential to prevent further complications.^108^ Maintaining good oral hygiene in children is also vital for promoting healthy tooth development. Following tooth eruption, the application of fluoride and pit-and-fissure sealants is highly recommended to protect against infectious diseases and support the proper maturation of permanent teeth.^66,109^

### Clinical management for tooth developmental anomalies

To ensure a comprehensive approach to the clinical management of tooth developmental anomalies, this consensus classifies management into three distinct stages: the temporary stage, encompassing the primary and mixed dentition stages, up to approximately 12 years of age; the transitional stage, spanning from the eruption of all permanent teeth (excluding the third molars) to adulthood, typically between 12 and 18 years of age; and the permanent stage, commencing after 18 years of age.^81^

**Fig. 6:**
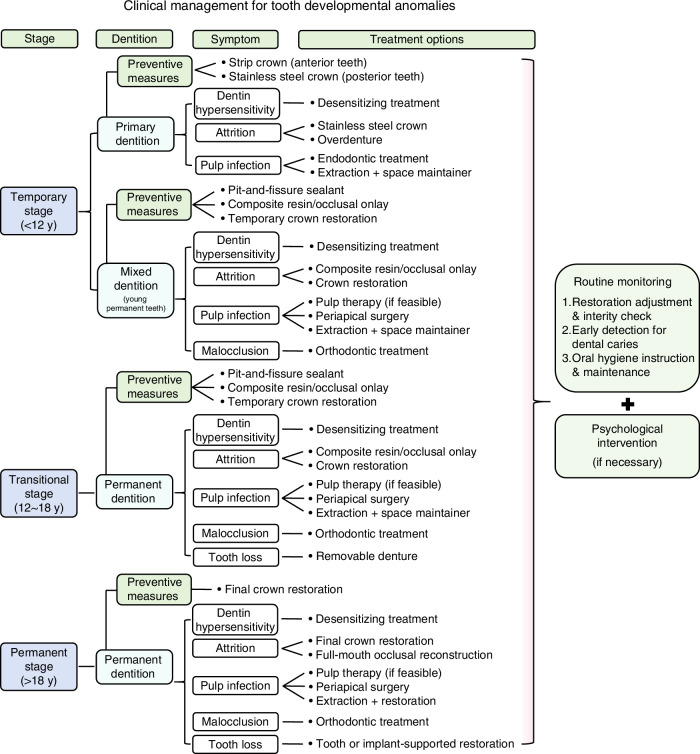
Summary of the clinical management for tooth developmental anomalies

The primary objectives of treatment include the elimination of infected tissues, preservation of tooth structure, reduction of tooth sensitivity, and maintenance of normal tooth function and maxillofacial development. Treatment strategies should be individualized based on the patient’s age, the severity of the condition, and specific clinical concerns. A personalized, multidisciplinary treatment plan should be formulated for each patient, integrating both oral health education and psychological support to optimize treatment outcomes.^92,110^ An overview of the treatment approaches is summarized in Fig. 6.

#### Temporary stage

During this stage, the primary objectives are to preserve the morphology and function of maxillofacial tissues while supporting their normal development.^82,83,111^ Effective management of infections, including dental caries and periapical lesions, is critical to preventing further complications. Additionally, efforts should be directed toward minimizing the risks of developing malocclusion and temporomandibular joint disorders (TMD). A comprehensive, multidisciplinary approach, incorporating expertise from endodontics, prosthodontics, orthodontics and periodontics, is essential to addressing the diverse clinical needs of patients.^111^

The treatment of primary dentition primarily aims to establish favorable conditions for the eruption of permanent teeth while supporting the development of the maxillofacial structures and temporomandibular joint (TMJ).^81^ In conditions such as AI and DGI, primary teeth may exhibit significant wear, necessitating an individualized prosthetic approach based on the severity of attrition.^110^ For primary teeth with normal morphology and minimal wear, anterior strip crowns and posterior stainless steel crowns are recommended as a preventive measure to preserve hard tissue and prevent tooth fracture.^112^ In cases where attrition is present, tooth preparation should be minimized or entirely avoided to preserve the remaining tooth structure.^113^ For teeth with severe attrition, removable overdentures are the preferred option.^114^ Primary teeth affected by severe periapical infections should be extracted to prevent further damage to the underlying permanent tooth buds.^115^ Following extraction, space maintainers should be placed to prevent impaction of permanent teeth and reduce the need for unnecessary orthodontic intervention. These space maintainers must be periodically adjusted or removed as the permanent teeth erupt to ensure proper alignment and occlusal function.^116^

The management of mixed dentition focuses on preserving tooth structure, alleviating hypersensitivity, maintaining vertical dimension, and restoring dental esthetics.^81^ In cases necessitating dentures, modifications should be implemented to accommodate newly erupted permanent teeth.^114,117^ Immediately after the emergence of young permanent teeth from the soft tissues, pit-and-fissure sealants should be applied to minimize the risk of dental caries.^118,119^ Subsequently, temporary occlusal onlays (for posterior teeth) or composite resin (for anterior teeth) is recommended to prevent excessive attrition of erupting teeth prior to their establishment of functional occlusion.^23,120^ Once the occlusion is established and the CEJ becomes accessible, indirect restorations can be used to protect the entire crown.^121^ In cases with pulp infection due to deep caries or attrition, pulp therapy is recommended, provided that the root canals are radiographically identifiable and amenable to treatment. However, teeth with severe infections that cannot be managed through pulp therapy or periapical surgery should be extracted, followed by the placement of space maintainers to prevent malocclusion. The resulting edentulous space may subsequently be restored with prosthetic rehabilitation or reduced through orthodontic intervention.^122^ Orthodontic treatment is particularly advantageous for children with malocclusion; however, thorough assessment is crucial before initiation, particularly in patients with short tooth roots or dental mobility, such as those with DD-I.^123^ In such cases, the application of light orthodontic forces is recommended to minimize the risks of root resorption and premature tooth exfoliation.^123,124^ In addition, for disorders associated with reduced tooth harness, such as DGI, clear aligner systems are preferred over traditional brackets to minimize tensile stress on the teeth.^125^

Structural anomalies have a significant impact on the bonding efficacy of both direct and indirect restorations applied to affected teeth.^126,127^ Specifically, in hypocalcified AI, the enamel exhibits an elevated organic content, which can compromise the effectiveness of acid etching, impede the formation of micropores, and ultimately reduce bond strength.^128,129^ Studies have demonstrated that treating the enamel surface with 5% sodium hypochlorite for 60 seconds effectively decreases organic content, thereby enhancing adhesive performance and bond durability.^129,130^ Moreover, conditions such as DGI, DD, and certain subtypes of AI are characterized by altered dentin structure. These alterations include a reduced number and diameter of dentin tubules, as well as an increased density of peritubular collagen fibers.^7,86,87,131^ In AI patients, the prolonged exposure of dentin due to accelerated enamel wear frequently leads to dentin sclerosis.^7^ Such structural modifications hinder adhesive infiltration and resin tag formation, both of which are essential for achieving durable bonding.^132,133^ In managing these dentin substrates, the etch-and-rinse adhesive system has been shown to provide superior bond strength compared to self-etch systems, making it the preferred choice for these clinical scenarios.^134^

During dental treatment, children often experience nervousness and anxiety, particularly in response to lengthy and complex procedures, which can lead to reduced cooperation. In the temporary stage, younger patients typically exhibit limited compliance to further complicating treatment. In cases where multiple teeth require intervention and the child is unable or unwilling to cooperate, clinicians may consider treatment under general anesthesia as an viable option.^135^

#### Transitional stage

During the transitional stage, all permanent teeth, except the third molars, have erupted; however, their roots may not be fully developed, and some teeth may not yet have reached functional occlusion. This stage typically spans from approximately 12 to 18 years of age, during which the permanent dentition begins to establish occlusal function, while the jaws and maxillofacial structures continue to develop. At this stage, preventive or transitional treatment strategies, similar to those used in mixed dentition, can be applied.^117,121^ For teeth with a poor prognosis that require extraction, removable dentures should be fabricated to maintain masticatory function and support the ongoing development of the maxillofacial structures. Once the patient reaches adulthood, fixed prosthetic restoration, supported by either natural teeth or implants, can be placed to restore both masticatory function and facial esthetics.^136^

Clinical observations from our team indicate that the shell-like teeth with enlarged pulp chambers observed in DGI-III gradually undergo pulp obliteration with age. During the shell-like teeth stage, transitional temporary restorations should be initiated promptly. Following the eruption of permanent teeth, onlays or composite resin restorations are recommended to protect the occlusal surface, prevent attrition and subsequent pulp infection, and preserve space for future final restorations. In particular, composite resin restorations are recommended for anterior teeth, while temporary occlusal onlays are preferred for posterior teeth to achieve optimal occlusal morphology and enhance masticatory efficiency.^23^ Once pulp obliteration occurs, the final restoration can be placed.^137,138^ In cases where periapical lesions develop after pulp obliteration, root canal negotiation may not always be necessary. Instead, long-term monitoring or periapical surgery may be considered as viable management options.^82^

#### Permanent stage

At this stage, patients have reached adulthood and achieved stable occlusion. Treatment during this stage primarily focuses on maintaining oral health through proper oral hygiene instruction and regular monitoring to prevent dental caries and periodontal diseases.^82^ For patients experiencing dentin hypersensitivity, desensitizing treatments should be administered. Depending on the severity, composite resin restorations or crowns may be considered as treatment options.^139^ In cases of infected teeth, thorough debridement should be performed, followed by appropriate restorative procedures based on the extent of the tooth defects.^140^ Crown restorations are recommended for patients with conditions such as AI and DGI to prevent further complications.^140,141^ For individuals with abnormal vertical dimension due to severe tooth attrition, full-mouth occlusal reconstruction is necessary to restore both masticatory function and facial esthetics.^141,142^ In cases of tooth loss, functional and esthetic rehabilitation should be achieved through natural tooth-supported or implant-supported restorations to ensure long-term oral health and stability.^143^

The primary objective in managing morphological anomalies such as DI and taurodontism is to prevent infection while preserving vital pulp and hard tissues. If the function and esthetics of the affected teeth remain uncompromised, regular monitoring is recommended. Preventive filling is recommended for DI when there are no signs of pulp infection. If pulp infection is present, treatment options such as root canal treatment (RCT), pulpotomy, apexification, pulp revascularization, or placement of an apical mineral trioxide aggregate barrier should be considered, depending on the extent of infection and the stage of apical foramen development.^31,36,97^ Caution is advised when treating these teeth due to their complex root canal anatomy, which can complicate debridement and obturation.^31,36^ In cases where periodontal infection is present, scaling and root planning should be performed. If the affected tooth has a poor prognosis or when treatment is deemed unfeasible, extraction may be the most appropriate option.^31,36^

#### Oral hygiene instruction and maintenance

Patients with tooth developmental anomalies remain at a high risk of dental caries and periodontal diseases, even after completing treatment.^144,145^ Therefore, regular follow-up care during and after treatment is essential to ensure long-term oral health maintenance. Regular check-ups help detect and manage issues such as new carious lesions or restoration failure, including filling exfoliation. For conditions with an increased predisposition to periodontal diseases, reinforced oral hygiene instruction and consistent periodontal maintenance are critical in mitigating risks.^146^ A check-up interval of three to six months is generally recommended, with shorter intervals advised for patients with more severe conditions to ensure optimal monitoring and preventive care.

#### Psychological intervention

Tooth developmental anomalies not only compromise oral function and esthetics but also have a profound impact on patients’ mental health. These conditions can contribute to low self-esteem, social avoidance, anxiety, and depression, particularly in adolescents, who are more susceptible to the long-term effects of masticatory dysfunction and esthetic concerns.^8^ For example, patients with AI exhibit higher levels of social avoidance and distress, as measured by the Social Anxiety Disorder (SAD) scale and the Fear of Negative Evaluation (FNE) scale.^147^ Consequently, psychological interventions play a critical role as adjunctive therapies in the management of tooth anomalies. These interventions aim to promote positive self-perception and greater acceptance of one’s appearance, thereby alleviating the psychological burden associated with these conditions.^148^ Effective implementation of such interventions may involve a combination of cognitive behavioral therapy, family-based interventions, and social skills training, ensuring a comprehensive approach to patient care.^149,150^ For specific approaches in this field, it is recommended to consult professional psychologists for detailed treatment plans.

## Summary

Standardized protocols for the diagnosis and management of tooth developmental anomalies continue to evolve. This expert consensus serves as practical guidance for clinicians, aiming to enhance diagnostic accuracy and treatment efficiency. Additionally, it underscores the importance of advancing research, improving early screening of diseases, and promoting precise treatment strategies to enhance patients’ oral health-related quality of life. Through multidisciplinary collaboration and clinical practice, diagnostic and treatment systems can be further optimized, ensuring comprehensive management and improved long-term outcomes for affected individuals. Future research on tooth developmental anomalies should focus on identifying unknown risk factors and genetic causes, as well as uncovering the underlying mechanisms. These insights are essential for advancing etiological treatment strategies. Additionally, well-designed clinical trials are urgently needed to compare treatment options and generate high-quality evidence to support clinical decision-making.
